# Psychometric Testing of the Kansas City Cardiomyopathy Questionnaire 12 in A European Population

**DOI:** 10.1097/JCN.0000000000001187

**Published:** 2025-02-27

**Authors:** Paolo Iovino, Hamilton Dollaku, Laura Rasero, Izabella Uchmanowicz, Rosaria Alvaro, Gianluca Pucciarelli, Ercole Vellone

**Affiliations:** **Paolo Iovino, PhD, RN** Assistant Professor, Health Sciences Department, University of Florence, Italy.; **Hamilton Dollaku, MS, RN** PhD Student, Department of Biomedicine and Prevention, University of Rome Tor Vergata, Italy; Fondazione Don Carlo Gnocchi, Florence, Italy.; **Laura Rasero, RN, MSN** Associate Professor, Health Sciences Department, University of Florence, Italy.; **Izabella Uchmanowicz, RN, PhD, FESC, FHFA** Professor, Department of Nursing and Obstetrics, Wroclaw Medical University, Poland.; **Rosaria Alvaro, MSN, RN, FAAN, FESC** Professor, Department of Biomedicine and Prevention, University of Rome Tor Vergata, Italy.; **Gianluca Pucciarelli, PhD, RN, FAHA** Associate Professor, Department of Biomedicine and Prevention, University of Rome Tor Vergata, Italy.; **Ercole Vellone, PhD, RN, FAAN, FESC** Professor, Department of Biomedicine and Prevention, University of Rome Tor Vergata, Italy; Faculty of Nursing and Midwifery, Wroclaw Medical University, Poland

**Keywords:** health status, heart failure, psychometrics, quality of life, reproducibility of results, surveys and questionnaires, validation study

## Abstract

**Introduction:**

The Kansas City Cardiomyopathy Questionnaire (KCCQ) is one of the most widely used instruments for measuring health-related quality of life of patients with heart failure (HF). However, its 12-item version (KCCQ-12) was not sufficiently tested in European populations, and its invariance in patients with reduced, midrange, and preserved ejection fraction has never been assessed.

**Aims:**

The purpose of this study is to examine the validity and reliability of the KCCQ-12 in a large cohort of Italian patients with HF and assess its measurement invariance across levels of ejection fraction.

**Methods:**

A total of 510 patients (mean age = 72 years, 58% males) completed the KCCQ-12 alongside other measures of depression, anxiety, quality of life, and HF symptom burden. Construct validity was assessed by means of a confirmatory factor analysis and by testing the association of the KCCQ-12 scores with clinical parameters (ie, ejection fraction and mortality at 12 months) as well as measures of anxiety, depression, symptom burden, and general quality of life. Multigroup confirmatory factor analysis was implemented to investigate invariance properties across those with reduced, midrange, and preserved ejection fraction. Omega and model-based internal consistency coefficients were computed to investigate internal consistency reliability.

**Results:**

The confirmatory factor analysis specified according to the original 4-factor model (ie, physical and social limitations, symptom frequency, and quality of life) yielded satisfactory fit indices (root mean square error of approximation = 0.053, comparative fit index = 0.98). Moderate-to-high correlations were found between the KCCQ-12 and the mental and physical component scores of the Short Form 12, as well as other conceptually related constructs, indicating adequate facets of construct validity. Multigroup confirmatory factor analysis tested across patients with different ejection fraction groups, established that the KCCQ-12 was invariant at the partial scalar level. Omega and model-based internal consistency coefficients were adequate, ranging from 0.75 to 0.90 for the subscales, and 0.94 for the whole scale, confirming strong internal consistency reliability.

**Conclusions:**

The KCCQ-12 demonstrated satisfactory psychometric properties in our European sample of patients with HF, providing evidence to support its use in practice and further research.

## Introduction

With more than 60 million individuals affected globally, heart failure (HF) has become a global health priority for all countries.^1^ Caused by cardiac injury and classically seen as the final stage of several cardiomyopathies, HF is characterized by a constellation of burdensome symptoms including dyspnea and fatigue, which significantly interact and ultimately compromise the quality of life of patients.^2^

Enhancing quality of life is a key goal of HF treatment together with prolonging survival.^3^ However, patients with HF still experience significant challenges that greatly impact their quality of life, as consistently reported by patients themselves.^4^ As a result, prognosis, which is an important correlate of quality of life, is adversely affected.^5^

In recent decades, there have been calls to integrate measurements of health-related quality of life (HR-QOL) into HF routine care and research. However, this self-report construct is challenging to capture because of its multidimensional nature. The Kansas City Cardiomyopathy questionnaire 12 (KCCQ-12) is one of the most recently used tools to measure the HR-QOL of patients with HF. Developed by Spertus and Jones^6^ in 2015, this tool aimed to address the excessive length of its predecessor, the KCCQ-23,^7^ which, because of this feature, is not suitable for serial routine screenings. In the development of the KCCQ-12, Spertus and Jones^6^ only considered to retain a few items on the domains that measure patients' health status, that is, physical limitation, symptom frequency, quality of life, and social limitation, consequently excluding the self-efficacy scale.^6^

Since its primary validation,^6^ the KCCQ-12 has demonstrated excellent validity and reliability properties across different patient populations affected by HF. For example, Dos Reis et al^8^ found that the scale has satisfactory psychometric properties on a sample of 124 patients with HF, whereas Albarrati et al^9^ tested the KCCQ-12 in Arabic patients. In the same years, Borregaard et al^10^ and Sauer et al^11^ extended the validation in patients with aortic valve stenosis and hypertrophic cardiomyopathy, respectively, confirming that this instrument can be valid and reliable also for other cardiac disease populations. Cultural adaptation of this instrument is important because the perception, conception, and expression of quality of life in these patients can be influenced by the cultural context.

Despite the confirmed validity and reliability of the KCCQ-12 across various cultures, currently, there is a general lack of validations of this scale on European patients affected by HF. The study by Dos Reis et al,^8^ conducted in Portugal, is the only published evidence known to us. However, it lacks rigorous validity as the authors did not test the dimensionality of the scale before proceeding to other types of validity and reliability estimations.

Given that HF is highly prevalent in Europe, validations of the KCCQ-12 in this context are greatly needed to promote screenings. Another significant gap that needs to be addressed is the lack of studies that establish the measurement equivalence of HR-QOL dimensions among patients with varying levels of disease severity, such as ejection fraction. Without this confirmation, differences in HR-QOL among these groups may possibly be the result of biased measurements.

### Aims

The primary objective of the present study was to psychometrically test the KCCQ-12 on a cohort of European patients diagnosed with HF. The specific objectives are to (1) ascertain the comprehensibility of the Italian version of the KCCQ-12, (2) assess construct validity (encompassing structural validity, hypothesis testing, and cross-cultural validity), as well as criterion validity (including predictive validity), and (3) evaluate the internal consistency reliability.

## Methods

### Design

We used a cross-sectional design using the baseline data collected on participants of the MOTIVATE-HF study, which was conducted to assess the effectiveness of motivational interviewing to improve self-care in patients with HF. The MOTIVATE-HF enrolled 510 patients from several outpatient clinics situated in the North and Center Italy. Details on methods, procedures, and main results can be consulted elsewhere.^12–14^

### Study Setting and Sampling

Patients and their caregivers were enrolled in 3 outpatient and community settings across Italy. Both dyad members were instructed to fill out a sociodemographic questionnaire and a battery of instruments autonomously or with the help of the research assistants.

### Sample Size

Because this is a secondary analysis, we first verified the adequacy of the sample by conducting a root mean square error of approximation–based power calculation.^15^ Given a power of 0.80, a critical *α* of .05, the minimum sample size needed was 248 (degrees of freedom = 48). Second, given the same power and critical *α*, a sample size of 193 is sufficient to detect a small effect size (*ρ* = 0.2) in correlation analyses.

### Inclusion Criteria

To be eligible for the MOTIVATE-HF study, patients had to have a diagnosis of HF according to international guidelines, a New York Heart Association class of at least II, and inadequate self-care, assessed with the Self-Care of Heart Failure Index.^16^ Severe cognitive impairment, acute coronary heart disease event during the 3 preceding months, and living in a residential setting precluded enrollment.

### Instruments and Data Source

The variables and related instruments used for the MOTIVATE-HF study are described elsewhere.^17^ For this analysis, the following were considered.

#### Health-Related Quality of Life

HR-QOL was collected with the KCCQ-12 and the Short Form 12. KCCQ-12 is a self-report 12-item tool that measures the 4 domains of physical functioning, symptoms, social functioning, and quality of life. Each domain has a score on a 0 to 100 range, with higher score points indicating better health status. An overall summary score can also be extracted, where 25, 50, and 75 represent the cut-offs of very poor/poor, poor/fair, fair/good, and good to excellent, respectively. The standardized partial scores (ie, for each domain) are computed by the following formula: [(actual raw score − lowest possible raw score)/possible raw score range]∗100. For example, the standardized score for the physical limitations domain is the following: [(sum of the actual scores of the items − 3)/(18 − 3)]∗100. The overall summary score is obtained by averaging the standardized scores of the 4 domains.

The Italian version of the KCCQ-12 was created by taking the respective items from the KCCQ-23 version. Subsequently, to verify the comprehensibility and answerability of the answers of the KCCQ-12 items, a set of cognitive interviews was conducted. We adopted the “think aloud” approach, where a group of 10 patients was requested to give their feedback of each item. The responses were rated according to the categories formulated by French et al.^18^ All the participant responses were assigned category 1: “no significant problem identified, which confirmed that the items were comprehensible and answerable to all the participants.

The Short Form 12 is a 12-item tool that measures general quality of life. Two component scores can be extracted reflecting the physical and mental domains. The instrument has been validated in patients with cardiac diseases, exhibiting acceptable predictivity.^19,20^ The Short Form 12 mental and physical component summary scores were used to test the discriminant validity of the KCCQ-12.

#### Anxiety and Depression

Anxiety and depression were measured with the Hospital Anxiety and Depression Scale, an instrument consisting of 14 items (7 items are for the anxiety subscale and 7 for the depression subscale).^21^ Scores range from 0 to 21, with higher scores representing worse anxiety and depression. Supportive validity and reliability have been demonstrated in cardiac patients.^22,23^ The 2 subscale scores were used to test the convergent validity of the KCCQ-12.

#### HF Symptoms

The burden of HF symptoms were measured with the Heart Failure Somatic Perception Scale, an 18-item tool that measures how burdensome are 18 physical signs and symptoms of HF disease.^24^ Scores range from 0 to 90, with higher score points indicating greater symptom burden. The Heart Failure Somatic Perception Scale has demonstrated supportive psychometric properties in patients with HF.^25^ The total Heart Failure Somatic Perception Scale score was extracted to test the convergent validity of the KCCQ-12.

#### Sociodemographic and Clinical Information

We collected self-reported information on age, gender, marital status, and educational attainment. Clinical variables of severity of the disease (ejection fraction and New York Heart Association class) were extracted from the health documentation.

### Statistical Analysis

The statistical analyses were performed with IBM SPSS version 2.5^26^ and MPLUS v.8.9.^27^ The analytical strategy was approached with 4 steps.

First, we conducted an item analysis using means and standard deviations. We inspected violations of normality of the KCCQ-12 items; we adopted the recommendations by Tabachnick and Fidell,^28^ where normal deviations stem from absolute values greater than |1|. Only few items were above this cut-off. Corrected item-total correlations were computed to test item homogeneity, where values greater than 0.30 are considered supportive.

The second step was conducted to test the construct validity of the KCCQ-12. This measurement property encompasses structural validity, hypothesis testing, and cross-cultural validity. To test the structural validity, we adopted a confirmatory factor analysis model, because the items and corresponding latent factors have already been defined. We used the robust maximum likelihood estimator (MLR) to derive model parameters, because a few items were not normally distributed. The following fit indices were adopted to assess model fit: (a) root mean square error of approximation (values less than 0.08 and test of close fit for the associated confidence intervals of *P* > .05 indicate adequate fit); (b) comparative fit index (values greater than 0.90 indicate adequate fit); (c) Tucker-Lewis index (values greater than 0.90 indicate adequate fit), and standardized root mean square residual (values lower than 0.08 indicate adequate fit). The *χ*^2^ statistic was also reported, but because of its sensitivity to sample size, its result was not used to interpret model fit.^29^ In case the first factors showed substantial correlations (>0.60), we also planned to examine a second-order confirmatory factor analysis, in line with the theoretical postulations^6,7^ and to give more strength to the overall summary score of the instrument. Construct validity was also investigated via hypothesis testing. Specifically, we hypothesized that the partial scores and the overall summary KCCQ-12 scores were negatively associated with those of the Hospital Anxiety and Depression Scale anxiety and depression subscales, and the scores of the Heart Failure Somatic Perception Scale symptom burden. We also hypothesized that the KCCQ-12 scores were positively associated with the Short Form 12 scores and that the KCCQ-12 scores increase across ejection fraction levels. Cross-cultural validity (ie, measurement invariance) was assessed across individuals with reduced (≤40%), midrange (41%–49%) and preserved (≥50%) ejection fraction.^30^ The rationale beyond this decision lies in that there is a strong relationship between HR-QOL and ejection fraction in patients with HF both in cross-sectional^31^ and longitudinal analyses.^32^ This may lead to different interpretations of the HR-QOL construct across the 3 groups, and therefore bias in measurement. We adopted the multigroup confirmatory factor analysis framework in line with the approach recommended by Meredith.^33^ The following levels of invariance were assessed: (a) configural invariance (ie, same factorial structure across groups); (b) metric (ie, factor loadings equal across groups); and (c) scalar (ie, equivalence of intercepts across groups). The nested models were compared with the differences in comparative fit index and root mean square error of approximation, where Δcomparative fit index and Δroot mean square error of approximation <0.01 and <0.015 suggest preservation of equivalence.^34^ If this solution is not achieved, full invariance cannot be claimed. Nevertheless, it is equally possible to achieve partial scalar invariance by releasing specific parameters indicated by inspecting the modification indices (*P* < .01). According to Byrne et al^35^ partial scalar invariance is sufficient for performing meaningful comparisons across groups.

The third step was conducted to investigate the criterion validity. Specifically, we tested the predictive validity by estimating the risk of death over 12 months with hazard ratios (HRs) as a function of the KCCQ-12 scores. Five multivariate Cox proportional hazard regressions were fitted by entering the randomization group in the model to adjust for the confounding effect of the educational intervention.

The fourth step was conducted to investigate the internal consistency of the KCCQ-12 as part of reliability testing. Composite reliability (Omega) was computed for each factor, whereas the model-based internal consistency index was computed for the total scale. Values greater than 0.70 are considered adequate for both indices.

### Ethical Considerations

The MOTIVATE-HF study was approved by the Institutional Review Board of Tor Vergata University (Rome). All the participants gave verbal and written informed consent to participate. The study is registered at ClinicalTrials.gov under identification number NCT02894502

## Results

### Characteristics of the Sample

The description of the sample is reported in Table 1. In brief, patients (n = 510) had a mean age of 72 years (SD = 12.3); were mostly males (58%), married (62%), unemployed or retired (83.9%); and did not live alone (84.7%). According to the New York Heart Association class, the sample was distributed as follows: class II (61.4%), class III (31.4%), and class IV (6.5%). In addition, 27.1% of the sample had a reduced ejection fraction, 41.2% had a midrange ejection fraction, and 31.8% had a preserved ejection fraction. Except for the symptom frequency domain, which scored fair to good (58.9, SD = 25.9), the other HR-QOL domains scored poor to fair (range, 33.4–44.5). The overall HR-QOL reported was also poor (44.5, SD = 22.1).

**TABLE 1:** Sociodemographic Characteristics of the Sample (n = 510)

	M (SD) or n (%)
Age, y	72.4 (12.3)
Gender (male)	296 (58)
Civil status	
Single	24 (4.7)
Married	316 (62.0)
Divorced/separated	20 (3.9)
Widowed	150 (29.4)
Live alone (yes)	78 (15.3)
New York Heart Association class	
II	313 (61.4)
III	160 (31.4)
IV	33 (6.5)
Ejection fraction	
Reduced	138 (27.1)
Midrange	210 (41.2)
Preserved	162 (31.8)
KCCQ-12	
Physical limitations	33.4 (23.2)
Symptom frequency	58.9 (25.9)
Quality of life	43.3 (27.2)
Social interference	42.5 (24.7)
Overall summary score	44.5 (22.1)

Abbreviation: KCCQ-12, Kansas City Cardiomyopathy Questionnaire.

**TABLE 2:** Descriptive Statistics of the Items of the Kansas City Cardiomyopathy Questionnaire 12 (n = 510)

	n	Mean	SD	Sk	Ku	Corrected Item-Total Correlation
1. Heart failure affects different people in different ways. Some feel shortness of breath while others feel fatigue. Please indicate how much you are limited by heart failure (shortness of breath or fatigue) in your ability to do the following activities over the past 2 weeks.						
*a. Showering/bathing*	510	2.88	1.31	0.16	−1.01	0.811
* b. Walking 1 block on level ground*	510	3.21	1.35	−0.12	−1.17	0.749
* c. Hurrying or jogging (as if to catch a bus)*	510	1.92	1.34	1.63	1.87	0.508
2. Over the past 2 weeks, how many times did you have swelling in your feet, ankles, or legs when you woke up in the morning?	510	3.70	1.30	−0.60	−0.78	0.534
3. Over the past 2 weeks, on average, how many times has fatigue limited your ability to do what you wanted?	510	3.95	1.83	0.04	−1.09	0.765
4. Over the past 2 weeks, on average, how many times has shortness of breath limited your ability to do what you wanted?	510	4.48	1.92	−0.19	−1.19	0.744
5. Over the past 2 weeks, on average, how many times have you been forced to sleep sitting up in a chair or with at least 3 pillows to prop you up because of shortness of breath?	510	3.65	1.44	−0.58	−1.08	0.558
6. Over the past 2 weeks, how much has your heart failure limited your enjoyment of life?	510	3.18	1.18	−0.04	−0.89	0.716
7. If you had to spend the rest of your life with your heart failure the way it is right now, how would you feel about this?	510	2.29	1.25	0.64	−0.64	0.618
8. How much does your heart failure affect your lifestyle? Please indicate how your heart failure may have limited your participation in the following activities over the past 2 weeks.						
* a. Hobbies, recreational activities*	510	3.16	1.33	0.18	−0.75	0.760
* b. Working or doing household chores*	510	2.91	1.32	0.40	−0.56	0.823
* c. Visiting family or friends out of your home*	510	3.30	1.42	−0.05	−1.04	0.792

Abbreviations: sk, skewness; ku, kurtosis.

### Item Analysis

Table 2 presents the item means of the KCCQ-12 as well as the skewness and kurtosis values, and the corrected item-total correlations. The highest mean was on item 4, indicating that shortness of breath frequently limited the patient's ability to perform certain activities. The lowest mean was on item 1c, indicating that shortness of breath did not limit the patient's activities performed hastily. All skewness and kurtosis indices did not exceed |3|, indicating satisfactory univariate normality. Corrected item-to-total correlations exceeded 0.30, indicating strong association between each item and the total score of the scale of the HR-QOL construct. Two items (1c and 7) exhibited significant floor effects, with a distribution of responses of 33.9% and 36.5%, respectively. The remaining distribution of extreme responses lay all below 15%. Regarding the overall summary and the partial domain scores, there was no evidence of clustering of patients at the extreme scores (<15% of the sample).

### Construct Validity

#### Structural Validity

The confirmatory factor analysis model specified as a 4-factor solution yielded optimal fit indices: *χ*^2^ (50, N = 510) = 121.29, *P* ≤ .001; root mean square error of approximation = 0.053 (90% CI, 0.041–0.065; *P* [root mean square error of approximation < 0.05] = .330); comparative fit index = 0.98; Tucker-Lewis index = 0.97; standardized root mean square residual = 0.028. The standardized factor loadings were all significant and high (>0.50). The correlations between the first-order factors were also significant, and all were greater than 0.60. Accordingly, a model with a second-order factor was specified, which exhibited the same fit indices as the previous model (Figure).

**FIGURE:**
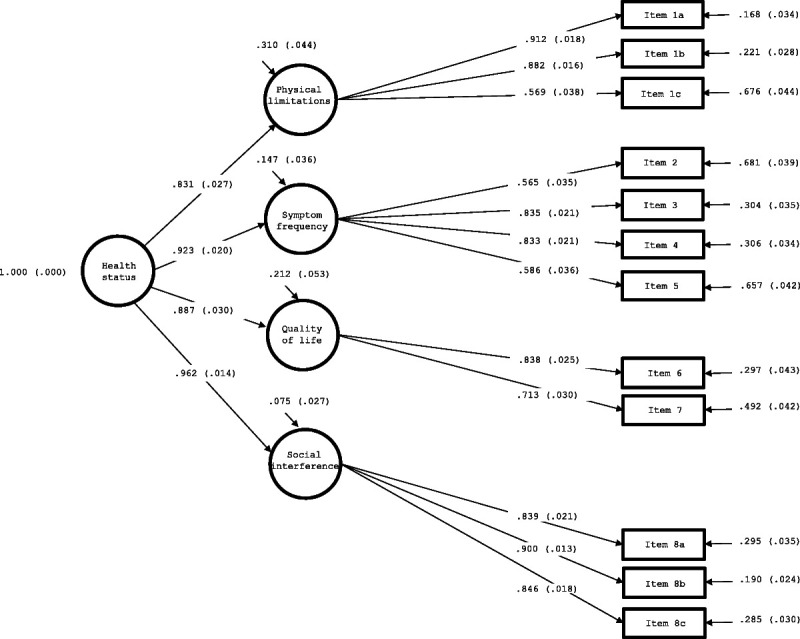
Confirmatory factor analysis of the Kansas City Cardiomyopathy Questionnaire 12 (KCCQ-12).

#### Hypothesis Testing

The KCCQ-12 scores were positively correlated with the Hospital Anxiety and Depression Scale anxiety and depression domains' scores and the Heart Failure Somatic Perception Scale (*P* < .001), confirming convergent validity. The KCCQ-12 and Short Form 12 scores were also positively correlated, confirming excellent construct validity (Table 3). There was a statistically significant difference in partial scores and overall summary score according to ejection fraction levels (*P* < .001). Bonferroni post hoc tests revealed that all the pairwise differences were significant except for the “symptom frequency” score, where the mean of the reduced ejection fraction showed no statistical difference from the mean of the midrange ejection fraction (*P* = .526).

**TABLE 3:** Correlations Between the Scores of the KCCQ-12 and Other Conceptually Related Variables (n = 510)

KCCQ Domains	Short Form 12 MCS	Short Form 12 PCS	Hospital Anxiety and Depression Scale Anxiety	Hospital Anxiety and Depression Scale Depression	Heart Failure Somatic Perception Scale
Physical limitations	0.378**	0.593**	−0.307**	−0.377*	−0.509**
Symptom frequency	0.466**	0.542**	−0.484**	−0.487**	−0.682**
Quality of life	0.530**	0.513**	−0.448**	−0.498**	−0.578**
Social interference	0.502**	0.605**	−0.422**	−0.477**	−0.611**
Overall summary score	0.539**	0.641**	−0.478**	−0.528**	−0.682**

Abbreviations: KCCQ-12, Kansas City Cardiomyopathy Questionnaire; MCS, mental component summary score; PCS, physical component summary score.

*P < .01; **P < .001.

#### Cross-Cultural Validity

Table 4 reports the results of the measurement invariance testing of the KCCQ-12 across the levels (ie, preserved, midrange, and reduced) of ejection fraction. Configural invariance was satisfactory with optimal fit indices. When constraints on factor loadings were imposed, full metric invariance was achieved (∆root mean square error of approximation = −0.004, ∆comparative fit index = −0.001). When constraints were imposed on intercepts, full scalar invariance was not achieved (∆RMSEA = 0.011, ∆comparative fit index = −0.022). An inspection of the modification indices indicated that items 1b, 1c, and 2 in the reducedejection fraction group, items 1a and 7 in the midrange ejection fraction group, and items 1a and 7 in the preserved ejection fraction group were not tenable. Upon releasing these intercepts, the fit improved and the ∆comparative fit index and ∆RMSEA fell below the recommended cut-off of 0.01 (∆root mean square error of approximation = 0.005, ∆comparative fit index = −0.008). Therefore, scalar invariance was achieved in its partial form. Table 5 reports the non-invariant intercepts in the 3 groups. Briefly, compared with their counterparts, those with reduced ejection fraction tended to systematically give lower scores in items 1b, 1c, and 2, whereas those with midrange and preserved ejection fraction tended to systematically give higher scores to item 1a and 7.

**TABLE 4:** Measurement Invariance Fit Indices of the KCCQ-12 Between Individuals With Preserved (n = 162), Midrange (n = 210), and Reduced (n = 138) Ejection Fraction

Model	*χ*^2^ (*df*)	Root Mean Square Error of Approximation (90% CI)	∆RMSEA	Comparative Fit Index	∆Comparative Fit Index	Decision
Configural invariance	225.700 (144)	0.058 (0.043–0.072)	–	0.973	–	Accept
Metric invariance	252.055 (168)	0.054 (0.040–0.068)	−0.004	0.972	−0.001	Accept
Scalar invariance	331.454 (192)	0.065 (0.053–0.077)	0.011	0.954	−0.022	Reject
Partial scalar invariance^a^	307.868 (187)	0.059 (0.049–0.071)	0.005	0.964	−0.008	Accept

Abbreviations: CI, confidence interval; KCCQ-12, Kansas City Cardiomyopathy Questionnaire; *df*, degree of freedom.

^a^Release of intercepts of items 1b, 1c, and 2 in the reduced ejection fraction group, items 1a and 7 in the midrange ejection fraction group, and items 1a and 7 in the preserved ejection fraction group.

**TABLE 5:** Non-invariant Intercepts Across Ejection Fraction Groups

	Reduced Ejection Fraction	Midrange Ejection Fraction	Preserved Ejection Fraction
Item 1a: How much are you limited by heart failure (shortness of breath or fatigue) in your ability to showering/bathing?	2.41	2.81^a^	3.20^a^
Item 1b: How much are you limited by heart failure (shortness of breath or fatigue) in your ability to walk 1 block on level ground?	2.71^a^	3.21	3.44
Item 1c: How much are you limited by heart failure (shortness of breath or fatigue) in your ability to hurry or jog (as if to catch a bus)?	1.51^a^	1.88	2.20
Item 2: How many times did you have swelling in your feet, ankles, or legs when you woke up in the morning?	3.70^a^	4.29	4.80
Item 7: If you had to spend the rest of your life with heart failure the way it is right now, how would you feel about this?	2.07	2.32^a^	2.27^a^

^a^Intercepts significantly different from the other groups.

### Criterion Validity

#### Predictive Validity

Cox proportional hazard models indicated that all the scores predicted death at 12 months except for the quality-of-life domain (physical limitation: HF = 0.98, *P* = .015; symptom frequency: HF = 0.98, *P* = .021; quality of life: HF = 0.99, *P* = .252; social interference: HF = 0.98, *P* = .028; overall summary score: hazard ratio = 0.97, *P* = .024).

### Reliability

#### Internal Consistency Reliability

When the internal consistency was investigated for each of the 4 KCCQ-12 domains, we obtained supportive Omega coefficients (physical limitations = 0.87, symptom frequency = 0.83, quality of life =0.75, social limitations = 0.90). The model-based internal consistency coefficient for the whole scale was 0.94.

## Discussion

In this large sample of patients with HF, we aimed to psychometrically test the KCCQ-12 using classical test theory. Our results indicate that this instrument has satisfactory validity and reliability. To the best of our knowledge, this is the first study that tested the KCCQ-12 with a rigorous psychometric approach and investigated the invariance properties of its measure across the ejection fraction levels.

In our analysis, we confirm that the KCCQ-12 administered on patients with HF has the same theoretical structure postulated by Spertus and Jones.^6^ Notably, in their validation study on 4168 patients, the authors only assumed the domains of the scale, because they never tested the factorial structure. Since then, only few studies validated the KCCQ-12 on patients with HF,^8,9^ and none of them adopted confirmatory analytical approaches. Despite this, there is evidence that, when the KCCQ-2 is tested in non-HF populations, the factorial structure can be different than the one originally postulated. For example, Borregaard et al^10^ studied a surgical population of patients affected by aortic valve stenosis and found that the instrument fit well with a 3-factor structure, which was also composed of different items, depending on the moment the tool was administered (ie, in the preoperative and postoperative phase). Intuitively, this is highly likely, because HF disease has a definite pattern of cardiac symptoms, which cannot be relevant for those with a different cardiac problem.

Our findings confirm that the KCCQ-12 has satisfactory construct validity via hypothesis testing, as indicated by the general significant and moderate to large correlations achieved between its partial and total scores and other specific psychological measures. Moreover, we confirmed that the KCCQ-12 has predictive validity because its scores can predict survival at 12 months. We also found that when compared across the groups of patients with different levels of ejection fraction, the KCCQ-12 is fully invariant at the configural and metric level, and partially invariant at the scalar level. These findings are important because the KCCQ-12 is frequently used to measure the HR-QOL of patients with varying ejection fraction levels, and this study supports the evidence that the KCCQ-12 scores can be compared across the 3 clinical levels of ejection fraction. Thus, researchers and clinicians can be confident that any estimated difference among these groups can be interpreted as a real difference on HR-QOL and not as a difference due to measurement bias.

The reliability of the KCCQ-12 was also supportive, which is consistent with the findings of the other validation studies.^8–10^ However, it is worth noticing that our results cannot be comparable because of improper use of the Cronbach *α* in these studies. More precisely, we went beyond the traditional Cronbach *α* for measuring the internal consistency of the domains, because this coefficient can yield biased estimations when used in models where the items have equal factor loadings in a factorial model.^36^ We also used a multidimensional coefficient to estimate the internal consistency of the whole scale, as opposed to others, who also used Cronbach *α* in this case, thus raising doubts about the real reliability of their data. Despite this, our results indicate that both the partial scores of the subscales and the overall summary score of the KCCQ-12 can be extracted reliably when we want to explore the multifaceted nature of the HR-QOL in patients with HF.

### Limitations

The present study has at least 2 limitations that are worth mentioning. The first is that we enrolled patients with inadequate self-care behaviors at baseline. Because there is strong evidence that self-care is positively associated with HR-QOL,^37,38^ it is likely that our sample had comparatively poorer HR-QOL than the general population, thus hindering generalizability to those with good health status. However, several studies have shown that self-care is low in HF populations.^39–41^ The second limitation is that, although we had sufficient longitudinal data available, the educational intervention administered in the MOTIVATE-HF precluded any test of reliability over time (ie, test-retest reliability).

## Conclusion

Our findings indicated that the KCCQ-12 has valid and reliable properties in our European sample of patients with HF, providing evidence to support its use in practice and further research. Future studies are warranted to examine other validity facets, such as test-retest and discriminant validity. Also, there is the need to understand whether the KCCQ-12 has the same strong psychometric properties in individuals affected by other cardiovascular diseases. Should it be confirmed, the KCCQ-12 use could be expanded in other contexts of care.

**What’s New and Important**

**Highlights of KCCQ-12:**

- Having a psychometrically validated assessment tool, which is fundamental to effective care of the patient with HF.
- Safety and usability of the tool in both clinical practice and research.
- Reinforcement of the confidence that health care professionals have in its effectiveness and, consequently, promotion of its increased use.

**Novelty:**

- The psychometric properties of the KCCQ-12 have been tested, and the instrument is valid for measuring HR-QOL in patients with HF.
- The use of the KCCQ-12 has significant benefits for both clinical practice and research.
